# The Role of Eco-Friendly Edible Insect Restaurants in the Field of Sustainable Tourism

**DOI:** 10.3390/ijerph17114064

**Published:** 2020-06-07

**Authors:** Jinsoo Hwang, Hyunjoon Kim, Ja Young Choe

**Affiliations:** 1 The College of Hospitality and Tourism Management, Sejong University, Seoul 143-747, Korea; jhwang@sejong.ac.kr; 2 The Department of Tourism Management, The College of Business Administration, Dong-A University, Busan 49236, Korea; 3 Faculty of Business Administration, University of Macau, Macau 853, China; jaceychoe@umac.mo

**Keywords:** edible insect restaurants, eco-friendly, green image, behavioral intentions, attitude, desire

## Abstract

The purpose of this study is to apply the concept of a green image in order to explore how to form behavioral intentions in the context of eco-friendly edible insect restaurants. This study analyzed 444 samples collected in South Korea in order to evaluate the theoretical model including 12 hypotheses. The data analysis results showed that a green image has a positive influence on attitude. In addition, attitude helps to increase desire, which in turn positively affects two sub-dimensions of behavioral intentions, such as intentions to use and word-of-mouth intentions.

## 1. Introduction

The world’s population is on a constant rise, and now it is approximately 7.7 billion, but will increase to about 9.7 billion by 2050 [1]. Such an increase in population causes severe food shortages because of limited resources [2]. Edible insects are receiving much attention as humans need new food sources to replace current food sources [3]. The Food and Agriculture Organization (FAO) also suggested that edible insects are the future food of mankind that can solve world hunger [4]. Edible insects are as high in protein, amino acids, and micronutrients, which are important nutrients for humans, as the meat of livestock [5]. Edible insects also have the advantage of not having a high-level entry barrier similar to that for livestock in terms of technology and monetary investment [6].

Green food can be defined as an organic and sustainable food in the restaurant industry [7], and previous studies have indicated that green food is regarded as pivotal for the sustainable development of the regional tourism [8]. In particular, in terms of the environmental aspect, edible insects aid to recycle animal waste, which leads to the protection of the environment [9]. In addition, Megido et al. also argued that compared to raising livestock, raising edible insects can reduce greenhouse gas, so it can be said that edible insects are green food [10]. According to Gössling and his colleagues [11], restaurant managers and the sustainable tourism service sector need to cooperate extensively in terms of environmental issues and global climate changes, and the industry should find ways to reduce carbon and waste for restaurant managers. In this sense, the eco-friendly role of edible insects is significantly related to the green image in the field of sustainable tourism since edible insect production not only has lower greenhouse gas emissions, but also higher feed-conversion efficiency because it requires less land and water when compared to livestock [12].

In recent years, consumers have a lot of interest in protecting the environment, so they try to purchase eco-friendly products/services, which fulfills their environmental needs [13]. For example, tourists actively enjoy involving themselves in sustainable food consumption including edible insects during their ecotourism holidays [12]. For this reason, restaurant companies make an effort to make their corporate image appear green to consumers [14]. If edible insect restaurants emphasize a green image based on their role of environmental protection, it will have a positive effect on consumer behavior. Although a green image of an edible insect restaurant is important in the context of sustainable tourism, there has been no research done on this subject.

To sum up, this study tried to explain how to form behavioral intentions in the context of eco-friendly edible insect restaurants. More specifically, this study examined the relationship between green image and attitude. In addition, this study investigated the effect of attitude on desire and two sub-dimensions of behavioral intentions including intentions to use and word-of-mouth intentions. In this situation, the results of this study will provide edible insect restaurant managers with important implications for developing effective green marketing strategies.

## 2. Literature Review

### 2.1. Eco-Friendly Edible Insect Restaurants

Although entomophagy, which means human consumption of insects, was first introduced by Bodenheimer [15], humans have been eating insects for thousands of years [16]. Currently, about two billion people around the world eat 1900 species of insects, such as crickets, buffalo worms, grasshoppers, ants, and cicadas as part of their food culture [17]. In addition, the market for edible insects in 2018 was approximately U.S. $400 million, and it is predicted to be approximately three times larger in 2023 [18]. According to Pliner and Hobden [19], food neophobia refers to a personal inclination to reluctance to try novel/unique foods. Thus, food neophobia and edible insects are highly correlated because edible insects can also seem disgusting to consumers. However, Olabi et al. [20] suggested that fear/negative emotions of novel/unique foods may gradually decrease with constant exposure. Therefore, in order to reduce the food neophobia of consumers, insect restaurant owners need to continuously promote edible insects, highlighting their merits.

These edible insects also play an important role in the restaurant industry. In the past, a small number of customers used edible insect restaurants, so they operated on a small scale. Nowadays, as more and more customers are looking for edible insect restaurants, their growth is exponential, and edible insect restaurants are operating even in five-star hotels [21]. For example, Linger restaurant, located in Denver, USA, uses insects as ingredients to provide a variety of menus to customers, and Sweet and Sour Crickets are the most popular [22]. In addition, Lardo, in Col. Condesa, an edible insect restaurant located in Mexico, is known for providing its customers with original menus, especially “huevo en torta”, which means eggs on cake made using insects. In fact, Mexico has a number of edible insect restaurants that provide an “exotic” experience to tourists [23]. In Thailand, “Insects in the Backyard” serves food using insects such as crickets, grasshoppers, worms, and beetles [24]. In particular, the restaurant emphasizes the nutritional aspects of insects, and suggests that insects play an important role in human future food. China has a long history of edible insects, especially insect restaurants in Yunnan Province have become popular [25]. Furthermore, there are many edible insect restaurants in England, Japan, New Zealand, and Taiwan, and sales are increasing [12].

In recent years, as consumers’ interest in environmental protection has increased in the restaurant industry, the eco-friendly role of edible insect restaurants has attracted attention [26]. Prior studies have consistently claimed that edible insects can play an important role in protecting the environment. First, it is widely known that many resources, such as grass and water, are used to raise livestock, which causes desertification [27]. On the other hand, edible insects are considered to be environmentally friendly because they require far fewer resources than livestock [28]. Second, greenhouse gas emissions from livestock have a negative impact on climate change [29], but edible insects have a very low level of greenhouse gas emissions, making a significant contribution to environmental protection [30]. Third, in terms of global warming potential (GWP), edible insects have lower levels of GWP compared to livestock such as beef and lamb [31].

In summary, edible insect restaurants are expected to play an important role in satisfying the eco-friendly needs of consumers, as edible insects are emerging as important new food sources that can replace traditional food sources in the environmental aspect. However, research on edible insect restaurants is insufficient.

### 2.2. Green Image

Human life has been enriched through several industrial revolutions, but on the contrary, these revolutions have caused environmental pollution [32]. In the late twentieth century, countries around the world recognized the seriousness of problems caused by environmental pollution, such as resource depletion and desertification, and governments have made efforts such as enacting laws on the protection of global environment [10]. As consumers’ awareness of environmental protection increases, they are willing to pay more to buy eco-friendly products that are more expensive than general products in order to protect the environment [33]. For this reason, companies are making a lot of effort in various forms, such as green marketing and green management to make their images green [34].

The concept of a green image refers to “a set of perceptions of a firm in a consumer’s mind that is linked to environmental commitments and concerns” [35]. Green includes the concept of natural environment and is also known as environmentally friendly or eco-friendly [36]. The green image of a company that satisfies consumers’ environmental needs plays a major role in enhancing trust in the company, consequently creating strong brand equity [34]. In addition, green image helps to maximize consumers’ intention to use and minimize consumers’ switch intentions [37]. More importantly, since a green image of a company has a symbolic meaning that can represent the overall characteristics of the company, the green image plays a critical role in differentiating it from other competitive brands [34].

Prior research has examined the outcome variables of a green image in diverse fields. For instance, Lee et al. [38] examined how a hotel’s green image affects behavioral intentions using 416 hotel guests. They found that the green image of the hotel favorably induces customers’ behavioral intentions. In addition, Yusof, Musa, and Rahman [39] developed a research model, which focused on the relationship between a green image and loyalty in the retail industry. Their data analysis results indicated that there is a positive relationship between a green image and loyalty. In other words, when people have a green image of a certain brand, they have a high loyalty to that brand. Martínez [35] also explored the effect of a green image on loyalty and identified that a green image positively affects loyalty. That is, people have high levels of brand loyalty if the brand has a green image.

### 2.3. Effect of Green Image on Attitude

First, this study proposes how a green image plays a role in forming attitude. Although many scholars have suggested the definition of attitude, the definition proposed by Ajzen [40] is the most frequently cited. The author emphasized the importance of attitude in order to explain consumer behavior through the theory of planned behavior (TPB), and defined attitude as “the degree to which a person has a favorable or unfavorable evaluation or appraisal of the behavior” [40]. In other words, attitude is a personal subject of an object or person, so the attitude is formed based on the values or beliefs that an individual seeks [41], which suggested that there is a positive association between green image and attitude. For instance, if consumers have strong beliefs about the protection of the natural environment, they will have a favorable attitude toward using eco-friendly products/services.

Existing studies have also supported the effect of green image on attitude. For example, Han, Yu, and Kim [42] explored the relationship between green image and attitude in the tourism industry, and they suggested that a green image is a vital predictor of attitude. That is, people have a favorable attitude to a certain brand when the brand has a green image. In addition, Hwang and Lyu [43] developed a research model in order to identify how a green image aids to enhance attitude in the airline industry, and they suggested that when consumers perceive a green image from a certain airline, they have a favorable attitude toward using that airline. Hwang and Kim [33] also investigated the role of green image in forming attitudes in the context of eco-friendly drone food delivery services. The authors showed that a green image helps to make consumer’s attitude favorable. Based on the theoretical and empirical studies discussed above, this study presents the following hypothesis.

**Hypothesis** **1** **(H1).** *Green image has a positive impact on attitude.*

### 2.4. Effect of Attitude on Desire and Behavioral Intentions

Desire is defined as “a state of mind whereby an agent has a personal motivation to perform an action or to achieve a goal” [44]. A particular behavior is formed by internal stimulation, which is known as the state of desire [44]. Desire is heavily affected by a positive or negative appraisal, which aids to form behavioral intentions [45]. For example, when a consumer makes a positive assessment of a certain product/service, they have a greater desire to use the product/service.

According to the model of goal-directed behavior (MGB), desire is a strong motivation for consumers to do a certain behavior they aim for and is shaped by the attitude they have [45], which suggested that attitude is an important factor influencing desire. Existing studies have confirmed the effect of attitude on desire. For example, Meng and Han [46] applied MGB to the field of bicycle tourism, and they found that attitude was found to bear a significant impact on desire. In other words, when consumers have a positive attitude toward bicycle tourism, they would have a high level of desire to do bicycle tourism. Kim and Preis [47] also examined the relationship between attitude and desire based on the extended MGB in the tourism industry. The authors indicated that when tourists’ attitudes toward using mobile devices for tourism-related purposes are good, they are more likely to have high levels of desire to use the devices. Based on the literature review above, this study proposed the following hypothesis.

**Hypothesis** **2** **(H2).** *Attitude has a positive impact on desire.*

Next, this study hypothesizes the relationship between attitude and behavioral intentions. Behavioral intentions are the likelihood that a person will attempt a particular behavior [40,48], and they consist of intentions to use and word-of-mouth intentions (e.g., Kim, Ng, and Kim, 2009; Maxham, 2001) [49,50]. Intentions to use can be defined as “the degree to which a person has formulated conscious plans to perform or not perform some specified future behavior” [51]. Consumers have intentions to use through a positive appraisal of products or services, which has a direct impact on a company’s sales [52]. In addition, word-of-mouth refers to “informal communication directed at other consumers about the ownership, usage or characteristics of particular goods and services and/or their sellers” [53]. The effect of word-of-mouth has more influence on consumers than the commercial advertisement of companies because people get information from acquaintances including family, friends, and relatives [54].

The relationship between attitude and behavioral intentions is theoretically supported by the theory of reasoned action (TRA) and the theory of planned behavior (TPB) [48,55], which indicated that attitude is an important factor that leads to behavioral intentions. The effect of attitude on behavioral intentions has been demonstrated in many fields. For instance, Alzahrani, Hall-Phillips, and Zeng [56] tried to find the relationship between attitude and behavioral intentions using the TRA, and they showed that when consumers have a favorable attitude toward using hybrid electric vehicles, they tend to have high levels of behavioral intentions. More recently, Yarimoglu and Gunay [57] employed the extended TPB in the green hotel industry. They showed that attitude is a significant predictor of behavioral intentions.

**Hypothesis** **3** **(H3).** *Attitude has a positive impact on intentions to use.*

**Hypothesis** **4** **(H4).** *Attitude has a positive impact on word-of-mouth intentions.*

### 2.5. Effect of Desire on Behavioral Intentions

According to MGB, desire to take a particular action is a significant predictor of behavioral intentions [58]. Empirical studies have also supported the argument. For example, Han, Lee, and Kim [59] developed a research model in order to find the relationship between desire and behavioral intentions. They showed that desire helps to enhance behavioral intentions. In addition, Hwang and Kim [33] examined the relationship between desire and behavioral intentions using in the context of drone food delivery services. They found that when consumers have high levels of desire to use drone food delivery services, they are more likely to use the services. Hwang, Cho, and Kim [60] also tried to investigate how desire affects behavioral intentions in the context of eco-friendly drone food delivery services. They suggested that desire plays an important role in the formation of behavioral intentions. Based on the theoretical and empirical backgrounds, the following hypotheses are proposed.

**Hypothesis** **5** **(H5).** *Desire has a positive impact on intentions to use.*

**Hypothesis** **6** **(H6).** *Desire has a positive impact on word-of-mouth intentions.*

### 2.6. Research Model

**Figure 1:**
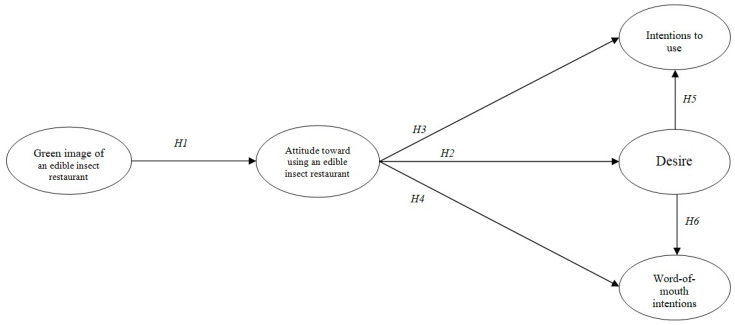
Proposed conceptual model.

Based on a total of six hypotheses, this study proposes the following research model (Figure 1).

## 3. Methodology

### 3.1. Measurement

First, a green image was measured with three items cited from Hwang and Kim [33] and Martínez [35]. Second, three items regarding attitude were cited from Ajzen [40] and Han and Hyun [61]. Third, desire was measured using three items adapted from Hwang and Kim [33] and Perugini and Bagozzi [58]. Fourth, in terms of behavioral intentions, three measurements for intentions to use were cited from Zeithaml, Berry, and Parasuraman [62], while word-of-mouth intentions were measured with three measurements borrowed from Hennig-Thurau, Gwinner, and Gremler [63].

All measurement items were measured using a seven-point Likert type scale (1 = strongly disagree; 7 = strongly agree). The survey was carefully reviewed by three expert groups including professors, graduate students, and restaurant employees in order to ensure content validity before finalizing the questionnaire, and they identified that there is high levels of content validity.

### 3.2. Data Collection

Data collection was performed using an online survey company system in South Korea. There were few edible insect restaurants in South Korea, so this study provided respondents with two newspaper articles and a video where anyone could easily understand the eco-friendly role of edible insect restaurants before beginning our survey. For example, the two newspaper articles and one video showed how to make insect foods and its important role in the protection of the environment. From the 6479 questionnaires distributed, 450 responses were collected. Responses with extreme answers and missing information were removed. As a result, 444 usable responses remained for further analysis. Prior studies including Hair et al. [64] and Weston and Gore [65] suggested that a sample size of 200 is satisfactory for performing confirmatory factor analysis (CFA) and structural equation modeling (SEM) with the maximum-likelihood estimation method. This implies that there was no problem with the representation of the sample.

## 4. Results

### 4.1. Profile of the Sample

The ratio of males and females in the sample was 50%. The mean age was 38.06 years, ranging from 20 to 59 years of age. The number of respondents in their 30s was the highest. In terms of monthly household income, 130 respondents (29.3%) answered that their income was between U.S. $1001 and U.S. $2000. In addition, 52.3% of the respondents (*n* = 232) were married, and 55.9% had a bachelor’s degree (*n* = 248).

### 4.2. Confirmatory Factor Analysis

Table 1 showed the results of confirmatory factor analysis. The results indicated that the overall fit of the measurement model was acceptable. The factor loadings were equal to or greater than 0.843 and all factor loadings were significant at *p* < 0.001. As shown in Table 2, the values of average variance extracted (AVE) were greater than 0.50 for all constructs, which is the threshold value [66]. Considering the high levels of factor loadings and also the values of AVE in the measurement model, convergent validity for all of the measurement items had been achieved [67].

As suggested by Fornell and Larcker [67], discriminant validity was evaluated by comparing the values of squared correlations between constructs and the values of AVE. The data analysis results showed that the values of AVE for each construct were higher than all of the squared correlations (*R*^2^) between a pair of constructs (Table 2), which suggested an acceptable discriminant validity. In addition, the values of composite reliability were greater than the 0.70 threshold [64], which showed that all of the measurement items were highly reliable and internally consistent.

### 4.3. Structural Equation Modeling

The SEM results showed that the proposed model fit the data well. Table 3 describes the SEM results with standardized coefficients and their t-values. The results showed that all six hypotheses were statistically accepted.

## 5. Discussion and Implications

The current paper was mapped out to explore the formation of behavioral intentions in the field of eco-friendly edible insect restaurants. A research model with a total of six hypotheses was tested using 444 samples collected in South Korea. The data analysis results indicated that there is a positive relationship between green image and attitude. Additionally, it was found that attitude was a critical predictor of desire. Furthermore, desire positively affects intentions to use and word-of-mouth intentions.

### 5.1. Theoretical Implications

First, the salient impact of green image on attitude was found, and it can be interpreted that when consumers perceive that edible insect restaurants are more likely to solve environmental problems, they are more likely to have a good attitude toward using them. As consumers become more environmentally conscious [68,69], research on green images becomes more common. For instance, the concept of green image was applied at airlines, café, drone food delivery services, and hotels [33,43], which suggested that a green image of the product/service makes the consumer’s attitude favorable. In this respect, the result of this study is consistent with previous studies. However, unlike previous studies, this study applied the concept of green image and revealed the effect of green image on attitude for the first time in the field of edible insect restaurants, thereby providing important theoretical implications.

Second, the SEM results identified attitude as an important factor in the formation of desire. That is, when consumers have a favorable attitude toward using edible insect restaurants, their desire of using the restaurants is strong. The relationship between attitude and desire was theoretically supported by the MGB [45], and also the existing literature has consistently confirmed the relationship (e.g., Hwang, Kim et al., 2019; Kim and Preis, 2016; Meng and Han, 2016) [33,46,47], which indicated that attitude plays a vital role in the formation of desire. In addition, our analysis identified the prominent influence of attitude on two sub-dimensions of behavioral intentions including intentions to use and word-of-mouth intentions. That is, consumers tend to dine out at edible insect restaurants and say positive things about them to others when they have a positive attitude. The theoretical evidence for the relationship between attitude and behavioral intentions is through TRA and TPB [48,55]. In addition, prior research has verified the relationship in many different fields (e.g., Alzahrani et al., 2019; Yarimoglu and Gunay, 2019) [56,57]. In this regard, the significant theoretical implication of this study is that we confirmed and extended the current literature by empirically identifying the effect of attitude on desire, intentions to use, and word-of-mouth intentions in the context of edible insect restaurants.

### 5.2. Managerial Implications

First, the current study confirmed that a green image of edible insect restaurants induces a positive attitude toward using them, which in turn positively affects desire and two sub-dimensions of behavioral intentions. The findings suggested the importance of green image in the context of edible insect restaurants. In fact, many restaurant companies are making an effort to impress consumers with their green image. For instance, Starbucks, the world’s largest coffee brand, will stop using disposable plastic straws by 2020 and replace them with paper or compostable plastic straws [70]. In addition, McDonald’s produces more than 3000 tons of coffee peel each year during roasting. McDonald’s plans to reduce the negative environmental impact by using the coffee peel as a vehicle material with Ford [71]. The efforts of these companies play an important role in delivering their green image to consumers. As explained earlier, edible insects are greener food sources than livestock when comparing greenhouse gas emissions and GWP [28,29]. Thus, if edible insect restaurant owners emphasize the eco-friendly aspects of edible insects using advertising, which is considered a significant means that create a green image (e.g., Ankit and Mayur, 2013; Yoon and Kim, 2016) [72,73], the consumers would have a favorable attitude toward using them. Furthermore, as the results of our data analysis indicated, consumers are more likely to have a high level of behavioral intentions toward edible insect restaurants.

Second, from a sustainable tourism, destination marketing organizations can emphasize the green role of edible insect restaurants for their marketing strategies. It is important to educate tourists that visiting an edible insect restaurant can reduce carbon emissions, maintain sustainability from the perspective of food miles, and support the region’s growing sustainable agriculture movement. It is recommended to provide menu books in an edible restaurant with a carbon footprint so that tourists can recognize to what extent they contribute to reduce the total greenhouse gas by their food choice. In addition, it is suggested that photos and promotional images include local farmers who sustain ways of life with growing edible insects to enhance the image of sustainability in a destination.

## 6. Limitations and Future Research

There are some limitations that provide opportunities for future research. First, the data collection was performed only in South Korea. Therefore, it is recommended that generalizing our study findings to other regions should be carefully done. Second, since edible insect restaurants are not activated in South Korea, the respondents in this study did not actually use the restaurants. In order to overcome this issue, two newspaper articles and a video, which clearly explain edible insect restaurants, were given to respondents before the start of the survey. However, it is necessary to collect data from other regions where edible insect restaurants are active in order to obtain data from people who actually use the restaurants. Third, eco-friendly behavioral intentions vary according to the demographic characteristics of consumers [74]. Therefore, future studies need to use demographic characteristics (e.g., gender and age) as a moderator.

## Figures and Tables

**Table 1:** Confirmatory factor analysis: items and loadings.

Construct and Scale Items	Standardized Loading ^a^
Green image of an edible insect restaurant	
An edible insect restaurant is more likely to be successful about its environmental protection.	0.843
An edible insect restaurant is more likely to solve environmental problems.	0.870
An edible insect restaurant is more likely to have a strong environmental reputation.	0.888
Attitude toward using an edible insect restaurant	
Unfavorable—Favorable	0.922
Bad—Good	0.886
Negative—Positive	0.929
Desire	
I desire to visit an edible insect restaurant.	0.936
My desire to visit an edible insect restaurant is strong.	0.970
I want to visit an edible insect restaurant.	0.961
Intentions to use	
I will dine out at an edible insect restaurant.	0.953
I am willing to dine out at an edible insect restaurant.	0.957
I am likely to dine out at an edible insect restaurant.	0.967
Word-of-mouth intentions	
I am likely to say positive things about an edible insect restaurant to others.	0.887
I am likely to recommend an edible insect restaurant to others.	0.980
I am likely to encourage others to dine out at an edible insect restaurant.	0.937
Goodness-of-fit statistics: χ^2^ = 211.179, df = 80, χ^2^ df ^−1^ = 2.640, *p* < 0.001, NFI = 0.977, CFI = 0.986, TLI = 0.981, and RMSEA = 0.061	

^a^ All factors loadings are significant at *p* < 0.001; df = Degree Freedom; NFI = normed fit index; CFI = comparative fit index; TLI = Tucker–Lewis index; and RMSEA = root mean square error of approximation.

**Table 2:** Descriptive statistics and associated measures.

Variables	No. of Item	Mean (SD)	AVE	(1)	(2)	(3)	(4)	(5)
(1) Green Image	3	4.56 (1.04)	0.752	0.901 ^a^	0.524 ^b^	0.563	0.526	0.580
(2) Attitude	3	4.06 (1.48)	0.833	0.275 ^c^	0.937	0.827	0.838	0.730
(3) Desire	3	3.67 (1.41)	0.914	0.317	0.684	0.969	0.840	0.828
(4) Intentions to Use	3	3.66 (1.42)	0.920	0.277	0.702	0.706	0.972	0.856
(5) Word-of-Mouth Intentions	3	3.78 (4.29)	0.875	0.336	0.533	0.686	0.733	0.954

SD = standard deviation; AVE = average variance extracted; ^a^ Composite reliabilities are along the diagonal; ^b^ Correlations are above the diagonal; ^c^ Squared correlations are below the diagonal.

**Table 3:** Standardized parameter estimates for structural model.

			Coefficients	*t*-Value	Hypothesis
H1 Green Image	→	Attitude	0.545	11.44 *	Supported
H2 Attitude	→	Desire	0.833	23.23 *	Supported
H3 Attitude	→	Intentions to use	0.149	4.19 *	Supported
H4 Attitude	→	Word-of-mouth intentions	0.123	2.18 *	Supported
H5 Desire	→	Intentions to use	0.833	21.44 *	Supported
H6 Desire	→	Word-of-mouth intentions	0.741	12.50 *	Supported
Goodness-of-fit statistics: χ^2^ = 261.048, *df* = 84, χ^2^ *df* ^−1^ = 3.108, *p* < 0.001, NFI = 0.972, CFI = 0.981, TLI = 0.976, and RMSEA = 0.069					

* *p* < 0.05; NFI = normed fit index; CFI = comparative fit index; TLI = Tucker–Lewis index; RMSEA = root mean square error of approximation.
